# Power and Sample Size Determination for the Group Comparison of Patient-Reported Outcomes with Rasch Family Models

**DOI:** 10.1371/journal.pone.0057279

**Published:** 2013-02-28

**Authors:** Myriam Blanchin, Jean-Benoit Hardouin, Francis Guillemin, Bruno Falissard, Véronique Sébille

**Affiliations:** 1 EA 4275, Biostatistics, Pharmacoepidemiology and Subjective Measures in Health Sciences, University of Nantes, Nantes, France; 2 EA 4360 Apemac, Université de Lorraine, Université Paris Descartes, Nancy, France; 3 INSERM 669, Université Paris-Sud and Université Paris Descartes, Paris, France; 4 Département de santé publique, Hôpital Paul Brousse, AP-HP, Villejuif, France; Politecnico di Torino, Italy

## Abstract

**Background:**

Patient-reported outcomes (PRO) that comprise all self-reported measures by the patient are important as endpoint in clinical trials and epidemiological studies. Models from the Item Response Theory (IRT) are increasingly used to analyze these particular outcomes that bring into play a latent variable as these outcomes cannot be directly observed. Preliminary developments have been proposed for sample size and power determination for the comparison of PRO in cross-sectional studies comparing two groups of patients when an IRT model is intended to be used for analysis. The objective of this work was to validate these developments in a large number of situations reflecting real-life studies.

**Methodology:**

The method to determine the power relies on the characteristics of the latent trait and of the questionnaire (distribution of the items), the difference between the latent variable mean in each group and the variance of this difference estimated using Cramer-Rao bound. Different scenarios were considered to evaluate the impact of the characteristics of the questionnaire and of the variance of the latent trait on performances of the Cramer-Rao method. The power obtained using Cramer-Rao method was compared to simulations.

**Principal Findings:**

Powers achieved with the Cramer-Rao method were close to powers obtained from simulations when the questionnaire was suitable for the studied population. Nevertheless, we have shown an underestimation of power with the Cramer-Rao method when the questionnaire was less suitable for the population. Besides, the Cramer-Rao method stays valid whatever the values of the variance of the latent trait.

**Conclusions:**

The Cramer-Rao method is adequate to determine the power of a test of group effect at design stage for two-group comparison studies including patient-reported outcomes in health sciences. At the design stage, the questionnaire used to measure the intended PRO should be carefully chosen in relation to the studied population.

## Introduction

Patient-reported outcomes (PRO) are important as endpoint in clinical trials and epidemiological studies. These outcomes comprise all self-reported measures by the patient regarding the patient’s health, the disease and its impact, or its treatment. They include health related quality of life, pain, patient satisfaction, psychological well-being, symptoms, treatment adherence/preference,… [1] PRO have first gained importance as secondary endpoints because they can be helpful to evaluate the effects of treatment on patient’s life or to study the quality of life of patient along with the disease progression to adapt the patient’s care. They can also be used as primary endpoint, especially in chronic diseases such as cancer [2], to compare two standard treatments with comparable survival outcomes or to help decision making. The deleterious impact of each treatment on patient’s quality of life can also be evaluated [3].

The singularity of PRO lies in the fact that the outcome, such as quality of life or wellness, cannot be directly observed. This particular outcome is defined as a latent variable. Generally, a questionnaire is the instrument that indirectly measures the latent variable and the responses of patients to items are further analyzed. Models from the Item Response Theory (IRT) link the probability of an answer to an item with item parameters and a latent variable. This theory has gained importance in Patient-Reported Outcomes area compared to the Classical Test Theory (CTT) where models are based on a score that often sums the responses to the items. IRT has shown advantages such as the management of missing data, the possibility to obtain an interval measure for the latent trait, the comparison of latent traits levels independently of the instrument, the management of possible floor and ceiling effects [4].

With the development of patient-reported outcomes in clinical research, guidelines were edited for construction, validation and administration of questionnaires [5]–[7]. However, the literature presents few references to the design stage. In particular, the sample size requirements when IRT is intended to be used for analysis of PRO seems to lack of theoretical work [8], [9]. When PRO are used as primary endpoint in a group comparison study, it is essential at the design stage to correctly determine the sample size to achieve the desired power for detecting a clinically meaningful difference in the future analysis. An inadequate sample size may lead to misleading results and incorrect conclusions. General recommendations on the sample size in the framework of education can be found. It should be highlighted that these recommendations are usually made without any theoretical justification. It is admitted that the sample size has to increase with the complexity of the model [10]: a number of 50 individuals was proposed for the simplest model of IRT, the Rasch model [11], a sample size of 200 respondents for the two-parameter logistic model has been suggested [12] and 500 examinees for the graded-response model [13]. Consequently, publications on health outcomes assessments make generally only few comments on the sample size determination as no analytical formula for the sample size exists.

It has been recently pointed out that the widely-used formula for the comparison of two normally distributed endpoints in two groups of patients was inadequate in the IRT setting [9]. Indeed, the power achieved by the tests of group effects using IRT modeling in a simulation study was lower than the expected power using the formula for normally distributed endpoints. Subsequently, Hardouin et al [14] have proposed a methodology to determine power related to sample size for PRO cross-sectional studies comparing two groups of patients in the framework of the Rasch model. The power determination depends on the difference between the expected means in the two groups (the group effect) and its standard error. The key point of the method is to estimate this standard error using the Cramer-Rao bound. This theoretical approach was first validated by simulation studies in some cases (small variance, appropriate questionnaire for the population under study) that may not reflect what is encountered in practice. Whether the method would perform as well in a large variety of situations often met in clinical and epidemiological studies remains unknown. As a matter of fact, the population of the study can have heterogeneous levels of the latent variable. Moreover, the PRO instrument might be more or less suitable for the population under study. Indeed, the items composing the instrument can be more or less relevant for the intended population of the study. For example, items from a disease-specific questionnaire (such as the QLQ-C30 [15] evaluating the quality of life of cancer patients) can be too difficult in a newly-diagnosed population in the sense that items specific to the disease can almost never be encountered in a population where the disease was recently detected, potentially before most of the symptoms appear. The measures provided by the PRO might not be reliable for all patients and the power could therefore be impacted by the choice of the questionnaire.

The purpose of this study was to validate the Cramer-Rao method for PRO cross-sectional studies comparing two groups of patients using the Rasch model. The impact of the variation of the variance of the latent variable (inter-patient heterogeneity regarding the latent variable) and of the distribution of the item parameters (appropriateness of the questionnaire for the population) on the proposed methodology has been studied by comparing the results of the Cramer-Rao method to the results of a simulation study.

## Methods

At the planning stage, the calculation of a sample size is usually based on a statistical test to detect a clinically meaningful effect at desired levels of type I and type II errors. In the case of the comparison of mean levels of PRO measures in two groups of patients, the widely-used formula for the comparison of two normally distributed endpoints may apply [16]. The formula assumes that the two groups are independent and that the variance of the endpoint 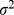 is common across the groups. The hypotheses for the two-sided test of comparison are defined as 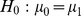 against 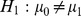, where 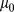 and 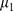 are the means of the endpoint in the first group and the second group respectively. The number of patients to be included in the first group 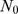 is determined by specifying an expected difference in the means of the PRO measures (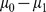) and the common variance (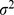) as well as the type I error (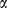) and the desired power (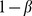) of the test.
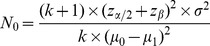
(1)where 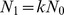 is the number of patients in the second group and 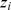 the 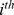 percentile of the standard normal distribution.

If this formula is adequate for manifest variables such as quality of life scores, it seems to incorrectly determine the sample size for latent variables [9] as it doesn’t take into account the uncertainty due to the estimation of the latent variable. So, this formula is not adapted for studies intending to use IRT models for the analysis.

### Sample Size and Power Determinations in IRT

#### The rasch model

In IRT, the link between a latent variable, that is the non-directly observable variable that the PRO instrument intends to measure (quality of life for example), and item parameters is modeled. Amongst the large family of IRT models, the Rasch model [17], [18] is largely used for dichotomous items in health sciences. It models the probability that a person 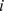 answers a response 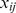 to an item 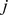 by a logistic model with two parameters, (i) the value of the latent variable of the person, 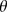 and (ii) the item parameter associated with the item 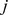, 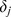. For a questionnaire composed of 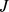 dichotomous items answered by 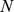 patients, the Rasch mixed model can be written as follows:
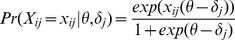


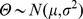
where 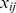 is a realization of the random variable 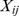 (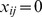 for the most defavorable response, 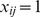 for the most favorable one). 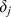 is also called the difficulty of item 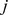. As the value of 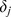 increases, the item is more and more difficult which means that patients are less and less likely to answer positively to the item. For example, an item “Does your health allows you to run an hour?” will be more difficult than an item “Does your health allow you to dress yourself?” if the positive answer is defined as “yes”. 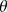 is a realization of the random variable 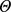, generally assumed to have a gaussian distribution. In this case, the parameters of the Rasch model can be estimated by marginal maximum likelihood (MML) [19]. A constraint has to be adopted to ensure the identifiability of the model. The nullity of the mean of the latent variable (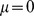) is often used for this purpose.

#### Power estimation using cramer-rao bound

In the design of a cross-sectional study for the comparison of two groups of patients in IRT, we are interested in the evaluation of a group effect, 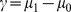, defined as the difference between the means of the latent variable in the two groups. Let 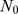 and 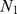 be the expected sample size in the first group and the second group respectively. To identify the model presented above, the constraint of the nullity of the mean of the latent variable 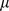 is adopted. The mean 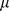 is the mean between 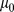 and 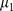, each of them weighted by the sample sizes 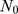 and 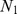. Consequently,
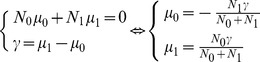



Let 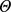 be a random variable representing the latent variable with normal distributions 
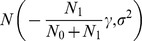
 and 
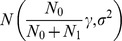
 in the first and the second group respectively. The variance of the latent trait 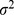 is assumed to be equal in the two groups. The mixed Rasch model including a covariate to estimate a group effect 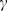 can be expressed as follows:
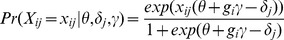


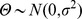
with 
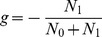
 in the first group and 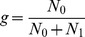 in the second group in order to meet the constraint of identifiability.

The sample size determination often relies on the Wald test to assess whether the group effect is significant. The following hypotheses are to be tested, 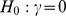 against 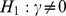. To perform the test, an estimate 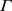 of 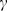 and its variance are required. The test statistic 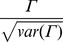 follows a normal distribution 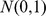 under 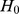. At the design stage, Hardouin et al. [14] proposed to use Fisher’s information and the Cramer-Rao (CR) boundary property to obtain an analytical formula for the standard error of 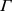. This method takes into account the characteristics of the questionnaire by using the parameters of the items to estimate the variance of the group effect. It also incorporates the uncertainty related to the estimation of the latent trait in the IRT model.

At the design stage, the item parameters are set to some planning expected values as well as 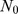, 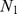, 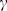 and 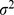. In addition, as the patient’s responses are not known, they should be determined. For each possible response patterns (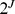 for binary response), the associated probability is computed for each group using the Rasch model, conditionally on the planned values of 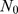, 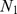, 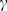 and 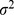. The expected frequency of each response pattern in each group is then determined [14]. The dataset created with the response patterns and their associated expected frequencies is analyzed using a mixed Rasch model including a group effect to estimate the variance of the group effect using CR and the power of the Wald test.

The expected power of the test of the group effect based on the Cramer-Rao bound (CR), 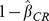, can be approximated by [14]:
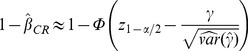
(2)with 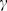 assumed to take a positive value, 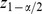 be the quantile of the standard normal distribution and 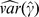 evaluated using Cramer-Rao bound.

The whole procedure has been implemented in the free Raschpower module accessible at http://rasch-online.univ-nantes.fr. This module determines the expected power of the test of the group effect based on the Cramer-Rao bound given the expected values of the sample size in each group (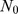 and 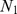), the group effect (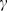), the variance of the latent variable (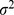) and the item parameters (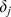) defined by the user.

### Simulation Study

To validate the Cramer-Rao method, the power determined with this method was compared to the power obtained by a simulation study, used as a reference.

#### Generation of data

Responses to 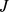 dichotomous items of two groups of patients were simulated using a mixed Rasch model where the latent variable has normal distributions 
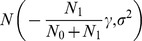
 and 
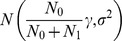
 in the first and the second group respectively.

**Figure 1:**
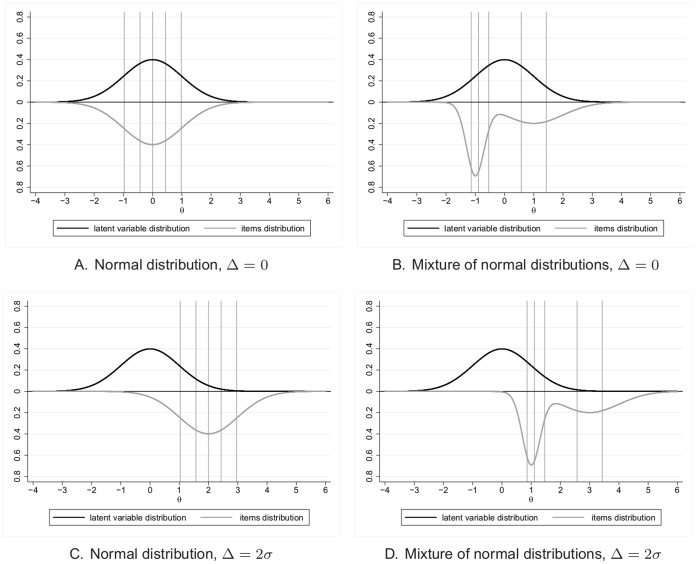
Distributions of items and latent variable for 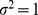 and 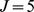. Vertical lines: values of the difficulties of the items.

To study the impact of the values of the item difficulties, the distribution of items could vary in two different ways according to the regularity of the spacing of the items and the gap between the mean of the latent variable and the mean of the items distribution. To obtain item difficulties that are quite regularly spaced, their values are set to the percentiles of a determined probability distribution. The normal distribution is used with the same mean and variance as the latent trait distribution. The questionnaire will therefore estimate the patients levels of quality of life with the same accuracy whatever the level of quality of life on the continuum of the latent trait as shown on Figure 1 (subfigure A). To obtain irregularly spaced item difficulties, an equiprobable mixture of two gaussian distributions was used. When the spacing is irregular, the estimates of the patients levels, of quality of life for example, will be more precise when difficulties are close to each other than when they are far apart from each other. We can see on Figure 1 (subfigure B) that the quality of life levels around −1 will be estimated more precisely than quality of life levels between −0.5 and 0.5. The case of irregular spacing of item difficulties is probably more encountered in practice than regular spacing.

The distribution of the items could be centered on the same mean as the latent trait or a gap, 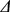, between the means of the latent trait and the mean of the item difficulties could be simulated. A positive gap is illustrated in Figure 1 (subfigures C and D). The latent variable distribution and the items distribution are then no more overlaid. The most difficult items of the questionnaire (on the right of the distribution) will be too difficult for the population. Hence, a very small part of the patients will respond positively to these items while most of the patients will respond positively to the easiest items (on the left of the distribution) leading to a floor effect. Due to this floor effect, the estimates of the patients levels will be less accurate on the left of the latent trait distribution (for poor levels of quality of life for example). In practice, a floor effect can occur when a disease-specific population answers to a generic questionnaire. For example, patients with serious physical impairment won’t be likely to answer positively to physical functioning items such as the ability to walk a block, to run or to climb stairs (example of items from the physical functioning of the generic questionnaire SF-36). On the opposite, a negative gap will lead to a ceiling effect as the items will be too easy for the studied population.

#### Parameters of the simulation study

The following values of the parameters were used in the simulation study:

- The number of individuals was equal in both groups (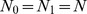) and could take the value 50, 100, 200, 300 or 500.
- The group effect (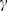) was equal to 0, 0.2, 0.5 or 0.8.
- The value of the variance of the latent trait (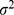) could be 0.25, 1, 4 or 9.
- The number of items (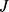) was 5 or 10.
- The item difficulties could come from a normal distribution 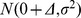 (quite regularly spaced) or from an equiprobable mixture of 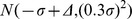 and 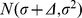 (irregularly spaced). The global mean of the latent variable was equal to 0. So, the distributions of items and latent traits were overlaid if the mean of item distribution was also equal to 0. The gap, 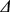, was defined as 0 (overlaid distributions), 1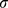, 2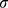. As the gap becomes larger, the items distribution departs more and more from the latent traits distribution and floor effect could occur more frequently. In the case of a normal distribution with a null 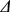, the questionnaire is assumed to be appropriate for the population without a floor effect and the items are quite regularly spaced.

The combination of all parameter values lead to 960 different cases. 1000 replications were simulated for each case.

#### Evaluated criteria

Each simulated dataset was analyzed with a mixed Rasch model including a covariate to estimate the group effect. A Wald test was then performed for assessing the significance of the group effect. For the simulations only, the type I error was estimated as the rate of rejection of the null hypothesis (null group effect) amongst datasets where the group effect was null (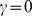). The confidence intervals of the type I error was computed as exact binomial proportion confidence intervals. The power of the test of group effect of the simulations, 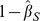, was estimated as the rate of significant tests amongst the simulations where the simulated value of 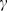 was not null. This result was compared with the estimated power using CR, 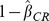 (eq. 2), computed with the Raschpower module of Stata [14]. As the estimation of 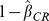 is based on the estimated value of the standard error of 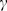, a good estimation of the power requires a good estimation of this standard error. Hence, the estimated value of the variance of the group effect in the simulation, 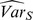, was compared with the estimated variance of the group effect using CR, 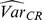.

## Results

### Estimation of the Variance of the Group Effect

Table 1 and table 2 show the estimated variance of group effect obtained either by simulations or using CR for all the values of parameters, for a group effect equals to 0 or 0.2 and 0.5 or 0.8, respectively. The estimations of the variance are close for both methods in general. As expected, the variance of the group effect decreases as 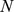 and 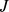 increase. Coherently, the variance of the group effect increases with the variance of the latent variable, 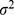. It slightly increases with the value of the group effect.

**Table 1:** Variance of the group effect estimated in the simulation study (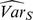) and using the Cramer-Rao’s bound (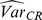) for different values of the group effect (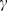), the sample size in each group (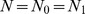), the variance of the latent variable (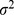), the number of items (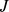), the spacing regularity of the items and the gap between the global mean of the latent variable and the mean of the distribution of the item difficulties (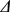).

			J = 5						J = 10					
			Normal distribution			Mixture of normal distributions			Normal distribution			Mixture of normal distributions		
			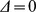	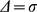	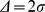	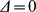	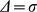	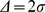	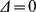	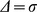	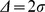	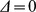	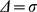	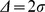
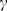	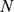	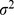	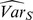 / 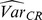	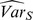 / 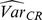	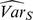 / 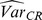	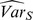 / 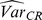	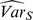 / 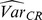	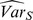 / 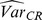	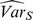 / 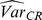	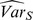 / 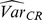	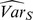 / 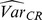	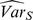 / 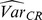	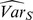 / 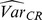	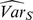 / 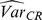
0	50	0.25	0.045/0.045	0.047/0.047	0.052/0.052	0.046/0.046	0.047/0.047	0.052/0.052	0.028/0.028	0.028/0.030	0.031/0.033	0.028/0.028	0.029/0.029	0.031/0.032
		1	0.082/0.082	0.088/0.089	0.113/0.113	0.085/0.085	0.088/0.088	0.107/0.107	0.062/0.062	0.065/0.067	0.078/0.081	0.063/0.063	0.065/0.066	0.075/0.077
		4	0.225/0.226	0.252/0.251	0.368/0.367	0.232/0.232	0.242/0.242	0.318/0.318	0.195/0.196	0.210/0.211	0.272/0.275	0.198/0.199	0.204/0.205	0.250/0.252
		9	0.458/0.459	0.520/0.521	0.782/0.777	0.466/0.467	0.493/0.493	0.645/0.641	0.413/0.413	0.447/0.447	0.589/0.595	0.417/0.418	0.432/0.433	0.520/0.518
	100	0.25	0.022/0.022	0.023/0.023	0.026/0.026	0.023/0.023	0.023/0.023	0.026/0.026	0.014/0.014	0.014/0.015	0.016/0.016	0.014/0.014	0.014/0.015	0.016/0.016
		1	0.041/0.041	0.044/0.044	0.056/0.056	0.042/0.042	0.044/0.044	0.053/0.053	0.031/0.031	0.033/0.033	0.039/0.040	0.031/0.032	0.032/0.033	0.037/0.038
		4	0.113/0.113	0.126/0.126	0.184/0.183	0.116/0.116	0.121/0.121	0.159/0.159	0.097/0.098	0.105/0.105	0.136/0.137	0.099/0.099	0.102/0.102	0.125/0.125
		9	0.229/0.229	0.260/0.259	0.389/0.388	0.233/0.233	0.246/0.246	0.321/0.320	0.206/0.207	0.223/0.224	0.294/0.294	0.208/0.209	0.216/0.216	0.259/0.259
	200	0.25	0.011/0.011	0.012/0.012	0.013/0.013	0.011/0.011	0.012/0.012	0.013/0.013	0.007/0.007	0.007/0.007	0.008/0.008	0.007/0.007	0.007/0.007	0.008/0.008
		1	0.020/0.020	0.022/0.022	0.028/0.028	0.021/0.021	0.022/0.022	0.027/0.027	0.015/0.015	0.016/0.016	0.019/0.020	0.016/0.016	0.016/0.016	0.019/0.019
		4	0.056/0.056	0.063/0.063	0.092/0.092	0.058/0.058	0.061/0.061	0.079/0.079	0.049/0.049	0.052/0.052	0.068/0.068	0.049/0.050	0.051/0.051	0.062/0.062
		9	0.114/0.114	0.130/0.130	0.194/0.194	0.116/0.116	0.123/0.123	0.161/0.161	0.103/0.103	0.112/0.112	0.147/0.147	0.104/0.104	0.108/0.108	0.129/0.129
	300	0.25	0.007/0.007	0.008/0.008	0.009/0.009	0.008/0.008	0.008/0.008	0.009/0.009	0.005/0.005	0.005/0.005	0.005/0.005	0.005/0.005	0.005/0.005	0.005/0.005
		1	0.014/0.014	0.015/0.015	0.019/0.019	0.014/0.014	0.015/0.015	0.018/0.018	0.010/0.010	0.011/0.011	0.013/0.013	0.010/0.011	0.011/0.011	0.012/0.013
		4	0.038/0.038	0.042/0.042	0.061/0.061	0.039/0.039	0.040/0.040	0.053/0.053	0.032/0.032	0.035/0.035	0.045/0.045	0.033/0.033	0.034/0.034	0.042/0.042
		9	0.076/0.076	0.086/0.087	0.129/0.129	0.078/0.078	0.082/0.082	0.107/0.107	0.069/0.069	0.074/0.074	0.098/0.098	0.069/0.069	0.072/0.072	0.086/0.086
	500	0.25	0.004/0.004	0.005/0.005	0.005/0.005	0.005/0.005	0.005/0.005	0.005/0.005	0.003/0.003	0.003/0.003	0.003/0.003	0.003/0.003	0.003/0.003	0.003/0.003
		1	0.008/0.008	0.009/0.009	0.011/0.011	0.008/0.008	0.009/0.009	0.011/0.011	0.006/0.006	0.006/0.007	0.008/0.008	0.006/0.006	0.006/0.007	0.007/0.008
		4	0.023/0.023	0.025/0.025	0.037/0.037	0.023/0.023	0.024/0.024	0.032/0.032	0.019/0.019	0.021/0.021	0.027/0.027	0.020/0.020	0.020/0.020	0.025/0.025
		9	0.046/0.046	0.052/0.052	0.078/0.078	0.047/0.047	0.049/0.049	0.064/0.064	0.041/0.041	0.045/0.045	0.059/0.059	0.042/0.042	0.043/0.043	0.052/0.052
0.2	50	0.25	0.045/0.045	0.045/0.047	0.045/0.053	0.045/0.046	0.045/0.047	0.045/0.052	0.028/0.029	0.028/0.030	0.028/0.033	0.028/0.029	0.028/0.029	0.028/0.032
		1	0.082/0.082	0.089/0.089	0.113/0.113	0.085/0.085	0.088/0.088	0.107/0.107	0.062/0.062	0.065/0.067	0.078/0.081	0.063/0.064	0.065/0.066	0.075/0.077
		4	0.226/0.226	0.225/0.252	0.225/0.371	0.225/0.232	0.226/0.242	0.225/0.318	0.195/0.196	0.195/0.211	0.195/0.276	0.195/0.198	0.195/0.205	0.195/0.252
		9	0.458/0.457	0.458/0.519	0.458/0.783	0.458/0.466	0.458/0.492	0.458/0.644	0.413/0.413	0.413/0.449	0.413/0.591	0.413/0.418	0.413/0.432	0.413/0.519
	100	0.25	0.022/0.022	0.022/0.023	0.022/0.026	0.022/0.023	0.022/0.023	0.022/0.026	0.014/0.014	0.014/0.015	0.014/0.016	0.014/0.014	0.014/0.015	0.014/0.016
		1	0.041/0.041	0.044/0.044	0.056/0.056	0.042/0.042	0.044/0.044	0.053/0.053	0.031/0.031	0.033/0.033	0.039/0.040	0.032/0.032	0.032/0.033	0.037/0.038
		4	0.113/0.113	0.113/0.126	0.113/0.184	0.113/0.116	0.113/0.121	0.113/0.158	0.097/0.098	0.097/0.105	0.097/0.137	0.097/0.099	0.097/0.102	0.097/0.125
		9	0.229/0.229	0.229/0.259	0.229/0.389	0.229/0.233	0.229/0.246	0.229/0.320	0.206/0.206	0.206/0.224	0.206/0.294	0.206/0.208	0.206/0.216	0.206/0.259
	200	0.25	0.011/0.011	0.011/0.012	0.011/0.013	0.011/0.011	0.011/0.012	0.011/0.013	0.007/0.007	0.007/0.007	0.007/0.008	0.007/0.007	0.007/0.007	0.007/0.008
		1	0.020/0.020	0.022/0.022	0.028/0.028	0.021/0.021	0.022/0.022	0.027/0.027	0.015/0.015	0.016/0.016	0.019/0.020	0.016/0.016	0.016/0.016	0.019/0.019
		4	0.056/0.056	0.056/0.063	0.056/0.092	0.056/0.058	0.056/0.061	0.056/0.079	0.049/0.049	0.049/0.052	0.049/0.068	0.049/0.049	0.049/0.051	0.049/0.062
		9	0.114/0.114	0.114/0.130	0.114/0.194	0.114/0.117	0.114/0.123	0.114/0.161	0.103/0.103	0.103/0.112	0.103/0.147	0.103/0.104	0.103/0.108	0.103/0.129
	300	0.25	0.007/0.007	0.007/0.008	0.007/0.009	0.007/0.008	0.007/0.008	0.007/0.009	0.005/0.005	0.005/0.005	0.005/0.005	0.005/0.005	0.005/0.005	0.005/0.005
		1	0.014/0.014	0.015/0.015	0.019/0.019	0.014/0.014	0.015/0.015	0.018/0.018	0.010/0.010	0.011/0.011	0.013/0.013	0.011/0.011	0.011/0.011	0.012/0.013
		4	0.038/0.038	0.038/0.042	0.038/0.061	0.038/0.039	0.038/0.040	0.038/0.053	0.032/0.032	0.032/0.035	0.032/0.045	0.032/0.033	0.032/0.034	0.032/0.042
		9	0.076/0.076	0.076/0.086	0.076/0.129	0.076/0.078	0.076/0.082	0.076/0.107	0.069/0.069	0.069/0.075	0.069/0.098	0.069/0.069	0.069/0.072	0.069/0.086
	500	0.25	0.004/0.004	0.004/0.005	0.004/0.005	0.004/0.005	0.004/0.005	0.004/0.005	0.003/0.003	0.003/0.003	0.003/0.003	0.003/0.003	0.003/0.003	0.003/0.003
		1	0.008/0.008	0.009/0.009	0.011/0.011	0.008/0.008	0.009/0.009	0.011/0.011	0.006/0.006	0.007/0.007	0.008/0.008	0.006/0.006	0.006/0.007	0.007/0.008
		4	0.023/0.023	0.023/0.025	0.023/0.037	0.023/0.023	0.023/0.024	0.023/0.032	0.019/0.019	0.019/0.021	0.019/0.027	0.019/0.020	0.019/0.020	0.019/0.025
		9	0.046/0.046	0.046/0.052	0.046/0.078	0.046/0.047	0.046/0.049	0.046/0.064	0.041/0.041	0.041/0.045	0.041/0.059	0.041/0.042	0.041/0.043	0.041/0.052

**Table 2:** Variance of the group effect estimated in the simulation study (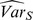) and using the Cramer-Rao’s bound (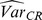) for different values of the group effect (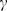), the sample size in each group (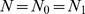), the variance of the latent variable (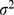), the number of items (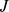), the spacing regularity of the items and the gap between the global mean of the latent variable and the mean of the distribution of the item difficulties (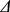).

			J = 5						J = 10					
			Normal distribution			Mixture of normal distributions			Normal distribution			Mixture of normal distributions		
			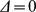	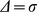	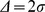	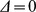	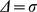	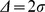	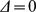	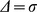	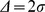	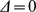	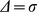	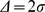
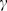	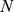	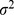	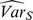 / 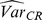	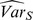 / 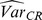	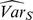 / 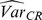	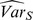 / 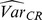	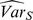 / 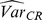	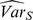 / 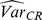	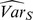 / 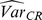	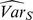 / 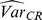	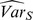 / 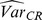	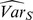 / 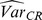	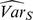 / 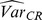	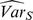 / 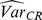
0.5	50	0.25	0.045/0.045	0.045/0.047	0.045/0.053	0.045/0.046	0.045/0.047	0.045/0.052	0.028/0.033	0.028/0.030	0.028/0.033	0.028/0.030	0.028/0.030	0.028/0.033
		1	0.082/0.082	0.089/0.089	0.113/0.114	0.085/0.085	0.089/0.089	0.107/0.107	0.062/0.063	0.065/0.067	0.078/0.081	0.063/0.064	0.065/0.066	0.075/0.077
		4	0.226/0.226	0.226/0.251	0.226/0.371	0.226/0.232	0.226/0.243	0.226/0.319	0.195/0.196	0.195/0.211	0.195/0.275	0.195/0.199	0.195/0.205	0.195/0.252
		9	0.458/0.459	0.458/0.520	0.459/0.777	0.459/0.466	0.458/0.492	0.459/0.642	0.413/0.413	0.413/0.449	0.413/0.591	0.413/0.417	0.413/0.432	0.413/0.521
	100	0.25	0.023/0.023	0.023/0.023	0.023/0.026	0.023/0.023	0.023/0.024	0.023/0.026	0.014/0.015	0.014/0.015	0.014/0.016	0.014/0.015	0.014/0.015	0.014/0.016
		1	0.041/0.041	0.044/0.044	0.056/0.057	0.043/0.043	0.044/0.044	0.053/0.054	0.031/0.031	0.033/0.033	0.039/0.040	0.032/0.032	0.033/0.033	0.038/0.038
		4	0.113/0.113	0.113/0.126	0.113/0.184	0.113/0.116	0.113/0.121	0.113/0.159	0.097/0.098	0.097/0.105	0.097/0.138	0.097/0.099	0.097/0.102	0.097/0.125
		9	0.229/0.229	0.229/0.259	0.229/0.389	0.229/0.233	0.229/0.247	0.229/0.322	0.206/0.207	0.206/0.224	0.206/0.294	0.206/0.209	0.206/0.216	0.206/0.260
	200	0.25	0.011/0.011	0.011/0.012	0.011/0.013	0.011/0.012	0.011/0.012	0.011/0.013	0.007/0.007	0.007/0.007	0.007/0.008	0.007/0.007	0.007/0.007	0.007/0.008
		1	0.021/0.021	0.022/0.022	0.028/0.028	0.021/0.021	0.022/0.022	0.027/0.027	0.015/0.015	0.016/0.016	0.019/0.020	0.016/0.016	0.016/0.016	0.019/0.019
		4	0.056/0.056	0.056/0.063	0.056/0.092	0.056/0.058	0.056/0.061	0.056/0.079	0.049/0.049	0.049/0.052	0.049/0.068	0.049/0.049	0.049/0.051	0.049/0.063
		9	0.114/0.114	0.115/0.130	0.115/0.194	0.114/0.117	0.115/0.123	0.115/0.161	0.103/0.103	0.103/0.112	0.103/0.147	0.103/0.104	0.103/0.108	0.103/0.130
	300	0.25	0.008/0.008	0.008/0.008	0.008/0.009	0.008/0.008	0.008/0.008	0.008/0.009	0.005/0.005	0.005/0.005	0.005/0.005	0.005/0.005	0.005/0.005	0.005/0.005
		1	0.014/0.014	0.015/0.015	0.019/0.019	0.014/0.014	0.015/0.015	0.018/0.018	0.010/0.010	0.011/0.011	0.013/0.013	0.011/0.011	0.011/0.011	0.012/0.013
		4	0.038/0.038	0.038/0.042	0.038/0.061	0.038/0.039	0.038/0.040	0.038/0.053	0.032/0.033	0.032/0.035	0.032/0.045	0.032/0.033	0.032/0.034	0.032/0.042
		9	0.076/0.076	0.076/0.086	0.076/0.130	0.076/0.078	0.076/0.082	0.076/0.107	0.069/0.069	0.069/0.075	0.069/0.098	0.069/0.069	0.069/0.072	0.069/0.086
	500	0.25	0.005/0.005	0.005/0.005	0.005/0.005	0.005/0.005	0.005/0.005	0.005/0.005	0.003/0.003	0.003/0.003	0.003/0.003	0.003/0.003	0.003/0.003	0.003/0.003
		1	0.008/0.008	0.009/0.009	0.011/0.011	0.009/0.009	0.009/0.009	0.011/0.011	0.006/0.006	0.007/0.007	0.008/0.008	0.006/0.006	0.007/0.007	0.007/0.008
		4	0.023/0.023	0.023/0.025	0.023/0.037	0.023/0.023	0.023/0.024	0.023/0.032	0.019/0.019	0.019/0.021	0.019/0.027	0.019/0.020	0.019/0.020	0.019/0.025
		9	0.046/0.046	0.046/0.052	0.046/0.078	0.046/0.047	0.046/0.049	0.046/0.064	0.041/0.041	0.041/0.045	0.041/0.059	0.041/0.042	0.041/0.043	0.041/0.052
0.8	50	0.25	0.046/0.046	0.046/0.048	0.046/0.053	0.046/0.047	0.046/0.048	0.046/0.053	0.028/0.034	0.028/0.030	0.028/0.033	0.028/0.032	0.028/0.030	0.028/0.033
		1	0.083/0.083	0.090/0.090	0.114/0.114	0.086/0.086	0.089/0.089	0.107/0.108	0.062/0.064	0.066/0.068	0.078/0.081	0.063/0.064	0.065/0.066	0.075/0.077
		4	0.226/0.227	0.226/0.253	0.226/0.368	0.226/0.232	0.226/0.243	0.226/0.320	0.195/0.196	0.195/0.212	0.195/0.278	0.195/0.199	0.195/0.205	0.195/0.252
		9	0.459/0.458	0.459/0.520	0.459/0.782	0.459/0.467	0.459/0.493	0.459/0.645	0.413/0.413	0.413/0.450	0.413/0.592	0.413/0.417	0.413/0.433	0.413/0.523
	100	0.25	0.023/0.023	0.023/0.024	0.023/0.027	0.023/0.023	0.023/0.024	0.023/0.026	0.014/0.016	0.014/0.015	0.014/0.016	0.014/0.015	0.014/0.015	0.014/0.016
		1	0.041/0.041	0.045/0.045	0.057/0.057	0.043/0.043	0.045/0.045	0.054/0.054	0.031/0.031	0.033/0.033	0.039/0.040	0.032/0.032	0.033/0.033	0.038/0.038
		4	0.113/0.113	0.113/0.126	0.113/0.185	0.113/0.116	0.113/0.121	0.113/0.159	0.098/0.098	0.098/0.105	0.098/0.138	0.098/0.099	0.098/0.103	0.098/0.126
		9	0.230/0.229	0.229/0.260	0.229/0.389	0.229/0.233	0.229/0.246	0.229/0.322	0.206/0.207	0.207/0.224	0.207/0.296	0.206/0.208	0.206/0.216	0.206/0.260
	200	0.25	0.011/0.011	0.011/0.012	0.011/0.013	0.011/0.012	0.011/0.012	0.011/0.013	0.007/0.007	0.007/0.007	0.007/0.008	0.007/0.007	0.007/0.007	0.007/0.008
		1	0.021/0.021	0.022/0.022	0.028/0.028	0.021/0.021	0.022/0.022	0.027/0.027	0.016/0.016	0.016/0.017	0.020/0.020	0.016/0.016	0.016/0.016	0.019/0.019
		4	0.057/0.057	0.057/0.063	0.057/0.092	0.057/0.058	0.057/0.061	0.057/0.080	0.049/0.049	0.049/0.053	0.049/0.068	0.049/0.050	0.049/0.051	0.049/0.063
		9	0.115/0.115	0.115/0.130	0.115/0.195	0.115/0.117	0.115/0.123	0.115/0.161	0.103/0.103	0.103/0.112	0.103/0.147	0.103/0.104	0.103/0.108	0.103/0.130
	300	0.25	0.008/0.008	0.008/0.008	0.008/0.009	0.008/0.008	0.008/0.008	0.008/0.009	0.005/0.005	0.005/0.005	0.005/0.005	0.005/0.005	0.005/0.005	0.005/0.005
		1	0.014/0.014	0.015/0.015	0.019/0.019	0.014/0.014	0.015/0.015	0.018/0.018	0.010/0.010	0.011/0.011	0.013/0.013	0.011/0.011	0.011/0.011	0.013/0.013
		4	0.038/0.038	0.038/0.042	0.038/0.061	0.038/0.039	0.038/0.040	0.038/0.053	0.033/0.033	0.033/0.035	0.033/0.046	0.033/0.033	0.033/0.034	0.033/0.042
		9	0.076/0.076	0.076/0.087	0.076/0.130	0.076/0.078	0.076/0.082	0.076/0.108	0.069/0.069	0.069/0.075	0.069/0.098	0.069/0.069	0.069/0.072	0.069/0.087
	500	0.25	0.005/0.005	0.005/0.005	0.005/0.005	0.005/0.005	0.005/0.005	0.005/0.005	0.003/0.003	0.003/0.003	0.003/0.003	0.003/0.003	0.003/0.003	0.003/0.003
		1	0.008/0.008	0.009/0.009	0.011/0.011	0.009/0.009	0.009/0.009	0.011/0.011	0.006/0.006	0.007/0.007	0.008/0.008	0.006/0.006	0.007/0.007	0.008/0.008
		4	0.023/0.023	0.023/0.025	0.023/0.037	0.023/0.023	0.023/0.024	0.023/0.032	0.020/0.020	0.020/0.021	0.020/0.027	0.020/0.020	0.020/0.020	0.020/0.025
		9	0.046/0.046	0.046/0.052	0.046/0.078	0.046/0.047	0.046/0.049	0.046/0.064	0.041/0.041	0.041/0.045	0.041/0.059	0.041/0.042	0.041/0.043	0.041/0.052

We note that the estimations of the variance for CR method are larger as compared to the simulation mostly when the gap is high (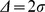) and 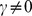. The highest overestimated values of the variance for CR are observed for low values of the sample size 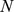 and of the number of items 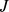, high values of the latent variable variance 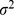 and a normal distribution of the items as compared to a mixture of normal distribution of items.

### Type I Error and Power of the Test of Group Effect

For the simulations, the type I error is well maintained to the expected value of 5% in almost all scenarios (results not shown). The type I error fluctuates between 2.6% (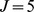, 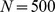, 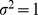, 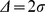, for a mixture distribution of the item difficulties) and 6.8% (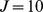, 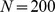, 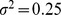, 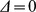, for a mixture distribution of the item difficulties). Amongst the 240 values of the type I error, only 9 confidence intervals at 95% of the estimated type I error don’t contain the expected value of 5%. None of the parameters seems to have an impact on the value of the type I error.

Table 3 and table 4 present the estimated values of the power obtained either by simulations or using CR for the values of all parameters for a questionnaire composed of 5 and 10 items, respectively. For simulations, the power was estimated by the rate of rejection of the null hypothesis amongst datasets where the group effect was not null (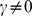). For all values of the simulation parameters, the estimated powers are close for each method (CR or simulations) when there is no gap. The difference between the powers obtained by simulation and using CR is around 0.003 in average and fluctuates between −0.034 (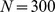, 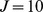, 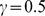, 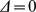, items normally distributed) and 0.059 (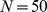, 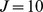, 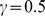, 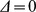, items normally distributed). As expected, the power increases as the sample size (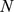) and the number of items (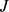) increase and decreases as the variance of the latent trait (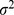) increases. It also increases with the group effect (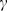).

**Table 3:** Power estimated in the simulation study (1−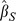) and using the Cramer-Rao’s bound (1−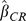) for different values of the sample size in each group (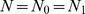), the group effect (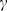), the variance of the latent variable (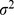), the spacing regularity of the items and the gap between the global mean of the latent variable and the mean of the distribution of the item difficulties (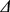).

			Normal distribution			Mixture of normal distributions		
			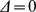	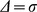	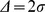	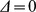	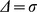	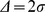
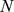	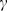	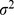	1− 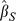 /1− 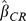	1− 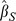 /1− 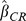	1− 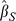 /1− 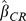	1− 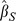 /1− 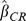	1− 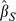 /1− 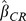	1− 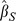 /1− 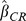
50	0.2	0.25	0.172/0.155	0.175/0.151	0.194/0.138	0.164/0.153	0.153/0.150	0.155/0.140
		1	0.116/0.104	0.110/0.099	0.085/0.086	0.113/0.102	0.096/0.099	0.095/0.089
		4	0.077/0.062	0.061/0.059	0.066/0.051	0.071/0.061	0.081/0.060	0.090/0.054
		9	0.073/0.048	0.056/0.046	0.055/0.041	0.067/0.048	0.058*/0.047	0.058/0.044
	0.5	0.25	0.645/0.652	0.685/0.635	0.658/0.586	0.652/0.645	0.696/0.633	0.680/0.589
		1	0.419/0.414	0.405/0.389	0.312/0.316	0.405/0.402	0.387/0.390	0.357/0.332
		4	0.171/0.182	0.170/0.168	0.171/0.127	0.186/0.178	0.177/0.172	0.177/0.141
		9	0.131/0.111	0.115/0.103	0.109/0.082	0.115/0.110	0.114*/0.106	0.112/0.091
	0.8	0.25	0.974/0.962	0.973/0.955	0.964/0.933	0.971/0.959	0.967/0.954	0.966/0.936
		1	0.796/0.794	0.794/0.762	0.672/0.660	0.772/0.780	0.758/0.764	0.685/0.682
		4	0.387/0.390	0.386/0.356	0.404/0.261	0.412/0.382	0.389/0.368	0.398/0.293
		9	0.211/0.218	0.233/0.198	0.224/0.146	0.215/0.215	0.250*/0.206	0.225/0.168
100	0.2	0.25	0.291/0.266	0.294/0.258	0.286/0.235	0.289/0.262	0.275/0.257	0.259/0.236
		1	0.188/0.166	0.171/0.156	0.147/0.132	0.167/0.161	0.169/0.157	0.140/0.137
		4	0.090/0.086	0.094/0.081	0.109/0.068	0.089/0.085	0.111/0.083	0.102/0.072
		9	0.068/0.062	0.075/0.059	0.069/0.051	0.073/0.061	0.069/0.060	0.069/0.054
	0.5	0.25	0.918/0.914	0.933/0.904	0.916/0.869	0.925/0.909	0.921/0.902	0.921/0.872
		1	0.693/0.694	0.686/0.661	0.537/0.557	0.667/0.679	0.659/0.661	0.547/0.580
		4	0.311/0.319	0.348/0.291	0.350/0.213	0.309/0.311	0.300/0.300	0.344/0.240
		9	0.166/0.180	0.159/0.164	0.164/0.123	0.171/0.178	0.177/0.170	0.182/0.140
	0.8	0.25	1.000/1.000	1.000/0.999	1.000/0.998	1.000/0.999	0.999/0.999	0.999/0.998
		1	0.975/0.976	0.972/0.966	0.925/0.919	0.968/0.972	0.963/0.966	0.921/0.932
		4	0.653/0.662	0.679/0.615	0.667/0.460	0.665/0.651	0.664/0.631	0.670/0.517
		9	0.391/0.386	0.373/0.348	0.378/0.249	0.388/0.381	0.383/0.364	0.379/0.291
200	0.2	0.25	0.450/0.472	0.452/0.457	0.467/0.416	0.484/0.464	0.491/0.455	0.475/0.420
		1	0.318/0.287	0.282/0.269	0.237/0.221	0.284/0.279	0.287/0.270	0.247/0.231
		4	0.118/0.132	0.137/0.123	0.133/0.097	0.122/0.129	0.109/0.126	0.124/0.106
		9	0.091/0.086	0.079/0.080	0.095/0.066	0.093/0.085	0.086/0.082	0.082/0.072
	0.5	0.25	1.000/0.997	0.999/0.996	0.999/0.992	1.000/0.997	0.996/0.996	0.998/0.992
		1	0.942/0.937	0.922/0.919	0.850/0.845	0.928/0.929	0.915/0.919	0.873/0.864
		4	0.532/0.558	0.577/0.513	0.545/0.378	0.559/0.546	0.577/0.528	0.525/0.427
		9	0.324/0.315	0.308/0.284	0.313/0.205	0.301/0.310	0.305/0.296	0.291/0.238
	0.8	0.25	1.000/1.000	1.000/1.000	1.000/1.000	1.000/1.000	1.000/1.000	1.000/1.000
		1	1.000/1.000	1.000/1.000	0.998/0.997	1.000/1.000	1.000/1.000	0.998/0.998
		4	0.924/0.920	0.921/0.890	0.920/0.752	0.933/0.913	0.913/0.901	0.904/0.810
		9	0.650/0.656	0.676/0.602	0.660/0.442	0.647/0.649	0.666/0.625	0.648/0.512
300	0.2	0.25	0.647/0.639	0.636/0.622	0.649/0.572	0.636/0.630	0.628/0.618	0.644/0.576
		1	0.413/0.402	0.377/0.377	0.306/0.308	0.397/0.391	0.383/0.378	0.355/0.323
		4	0.174*/0.177	0.189/0.163	0.193/0.125	0.175/0.173	0.176/0.167	0.176/0.138
		9	0.131/0.108	0.117/0.100	0.097/0.080	0.112/0.107	0.110/0.103	0.098/0.089
	0.5	0.25	1.000/1.000	1.000/1.000	1.000/1.000	1.000/1.000	1.000/1.000	1.000/1.000
		1	0.993/0.990	0.986/0.984	0.944/0.954	0.988/0.987	0.979/0.984	0.966/0.963
		4	0.728*/0.732	0.714/0.685	0.718/0.524	0.743/0.720	0.724/0.701	0.728/0.585
		9	0.462/0.440	0.456/0.397	0.441/0.284	0.428/0.434	0.445/0.415	0.451/0.332
	0.8	0.25	1.000/1.000	1.000/1.000	1.000/1.000	1.000/1.000	1.000/1.000	1.000/1.000
		1	1.000/1.000	1.000/1.000	1.000/1.000	1.000/1.000	1.000/1.000	1.000/1.000
		4	0.984*/0.985	0.988/0.974	0.982/0.898	0.988/0.982	0.988/0.978	0.985/0.935
		9	0.825/0.825	0.835/0.776	0.834/0.603	0.828/0.819	0.824/0.797	0.833/0.684
500	0.2	0.25	0.824/0.848	0.859/0.834	0.851/0.789	0.853/0.841	0.864/0.831	0.854/0.793
		1	0.587/0.599	0.546/0.566	0.458/0.470	0.600/0.584	0.585/0.567	0.469*/0.491
		4	0.278/0.265	0.271/0.242	0.259/0.180	0.263/0.259	0.271/0.250	0.246/0.201
		9	0.170/0.153	0.120/0.140	0.165/0.107	0.145/0.151	0.169/0.145	0.152/0.121
	0.5	0.25	1.000/1.000	1.000/1.000	1.000/1.000	1.000/1.000	1.000/1.000	1.000/1.000
		1	1.000/1.000	0.999/1.000	0.998/0.997	0.998/1.000	1.000/1.000	0.996*/0.998
		4	0.913/0.915	0.917/0.883	0.925/0.742	0.900/0.907	0.932/0.895	0.909/0.801
		9	0.660/0.647	0.672/0.593	0.639/0.435	0.647/0.639	0.626/0.615	0.634/0.504
	0.8	0.25	1.000/1.000	1.000/1.000	1.000/1.000	1.000/1.000	1.000/1.000	1.000/1.000
		1	1.000/1.000	1.000/1.000	1.000/1.000	1.000/1.000	1.000/1.000	1.000*/1.000
		4	1.000/1.000	0.999/0.999	0.998/0.987	0.999/0.999	0.999/0.999	0.999/0.994
		9	0.947/0.962	0.967/0.939	0.949/0.818	0.971/0.959	0.960/0.950	0.975/0.883

Results for a questionnaire composed of 5 items.

* The 95% confidence interval of the type I error does not contain the expected value of 5%.

**Table 4:** Power estimated in the simulation study (1−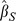) and using the Cramer-Rao’s bound (1−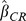) for different values of the sample size in each group (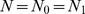), the group effect (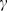), the variance of the latent variable (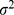), the spacing regularity of the items and the gap between the global mean of the latent variable and the mean of the distribution of the item difficulties (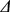).

			Normal distribution			Mixture of normal distributions		
			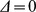	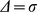	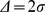	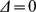	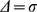	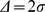
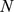	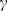	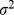	1− 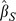 1− 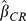	1− 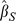 1− 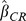	1− 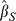 1− 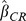	1− 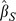 1− 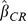	1− 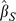 1− 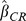	1− 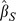 1− 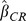
50	0.2	0.25	0.224/0.219	0.222/0.213	0.225/0.196	0.206/0.218	0.228/0.214	0.209/0.198
		1	0.133/0.123	0.129/0.118	0.109/0.105	0.118/0.122	0.125/0.119	0.133/0.108
		4	0.052/0.066	0.060/0.064	0.061/0.057	0.064/0.065	0.069/0.064	0.071/0.059
		9	0.077/0.050	0.064/0.048	0.069/0.045	0.061/0.049	0.056/0.049	0.067/0.046
	0.5	0.25	0.849/0.790	0.849/0.829	0.845/0.789	0.841/0.817	0.857/0.829	0.866/0.792
		1	0.513/0.513	0.493/0.488	0.425/0.420	0.513/0.508	0.472/0.495	0.421/0.437
		4	0.198/0.203	0.197/0.192	0.192/0.157	0.226/0.201	0.210/0.196	0.202/0.168
		9	0.115/0.119	0.142/0.112	0.121/0.095	0.129/0.118	0.114/0.115	0.120/0.103
	0.8	0.25	0.999/0.991	0.997/0.996	0.997/0.993	0.998/0.994	0.997/0.996	0.998/0.993
		1	0.899/0.887	0.878/0.868	0.801/0.803	0.886/0.884	0.888/0.874	0.822/0.820
		4	0.446/0.439	0.441/0.412	0.418/0.329	0.418/0.434	0.433/0.423	0.458/0.357
		9	0.273/0.237	0.225/0.222	0.257/0.179	0.243/0.235	0.230/0.229	0.248/0.197
100	0.2	0.25	0.401/0.393	0.414/0.381	0.400/0.349	0.385/0.391	0.420/0.381	0.422/0.352
		1	0.208/0.205	0.208/0.195	0.183/0.169	0.199/0.202	0.201/0.197	0.188/0.176
		4	0.086/0.093	0.098*/0.090	0.108/0.078	0.097/0.093	0.088/0.091	0.094/0.082
		9	0.072/0.064	0.084*/0.062	0.067/0.056	0.080/0.064	0.057/0.063	0.079/0.059
	0.5	0.25	0.988/0.984	0.987/0.985	0.988/0.975	0.989/0.985	0.988/0.985	0.984/0.977
		1	0.831/0.809	0.795/0.782	0.711/0.707	0.822/0.802	0.788/0.788	0.743/0.729
		4	0.361/0.359	0.376*/0.338	0.361/0.270	0.334/0.355	0.353/0.346	0.327/0.292
		9	0.217/0.195	0.204*/0.183	0.192/0.150	0.198/0.193	0.212/0.188	0.197/0.164
	0.8	0.25	1.000/1.000	1.000/1.000	1.000/1.000	1.000/1.000	1.000/1.000	1.000/1.000
		1	0.994/0.995	0.993/0.992	0.985/0.980	0.991/0.994	0.990/0.993	0.984/0.983
		4	0.720/0.725	0.723*/0.693	0.722/0.577	0.718/0.719	0.725/0.705	0.714/0.616
		9	0.399/0.421	0.422*/0.394	0.453/0.312	0.430/0.418	0.412/0.406	0.415/0.348
200	0.2	0.25	0.695*/0.671	0.682/0.654	0.663/0.609	0.641*/0.664	0.679/0.653	0.683/0.612
		1	0.364/0.363	0.356/0.345	0.281/0.296	0.362/0.356	0.331/0.347	0.303/0.307
		4	0.141/0.146	0.159/0.139	0.153/0.116	0.152/0.144	0.143/0.141	0.148/0.123
		9	0.095/0.091	0.087/0.087	0.095/0.075	0.102/0.090	0.100/0.088	0.124/0.080
	0.5	0.25	1.000*/1.000	1.000/1.000	1.000/1.000	1.000*/1.000	1.000/1.000	1.000/1.000
		1	0.984/0.980	0.973/0.974	0.939/0.945	0.973/0.978	0.980/0.974	0.951/0.953
		4	0.616/0.620	0.609/0.588	0.638/0.482	0.627/0.613	0.609/0.599	0.655/0.515
		9	0.341/0.343	0.327/0.321	0.343/0.256	0.352/0.341	0.365/0.331	0.354/0.284
	0.8	0.25	1.000*/1.000	1.000/1.000	1.000/1.000	1.000*/1.000	1.000/1.000	1.000/1.000
		1	1.000/1.000	1.000/1.000	1.000/1.000	1.000/1.000	1.000/1.000	1.000/1.000
		4	0.942/0.951	0.959/0.937	0.948/0.864	0.946/0.949	0.935/0.942	0.941/0.891
		9	0.696/0.702	0.703/0.667	0.691/0.549	0.692/0.698	0.700/0.683	0.709/0.602
300	0.2	0.25	0.838/0.838	0.831/0.824	0.831/0.785	0.835/0.832	0.822*/0.822	0.839/0.787
		1	0.481/0.505	0.490/0.483	0.419/0.416	0.494/0.496	0.518/0.484	0.447/0.430
		4	0.231/0.198	0.170/0.187	0.191/0.153	0.188/0.195	0.178/0.190	0.204/0.164
		9	0.096/0.116	0.125/0.110	0.102/0.093	0.109/0.115	0.130/0.112	0.104/0.100
	0.5	0.25	1.000/1.000	1.000/1.000	1.000/1.000	1.000/1.000	1.000*/1.000	1.000/1.000
		1	0.997/0.998	0.998/0.998	0.994/0.992	0.998/0.998	0.998/0.998	0.992/0.994
		4	0.806/0.792	0.803/0.762	0.781/0.650	0.821/0.786	0.795/0.773	0.794/0.688
		9	0.444/0.478	0.479/0.449	0.467/0.358	0.471/0.475	0.490/0.462	0.492/0.398
	0.8	0.25	1.000/1.000	1.000/1.000	1.000/1.000	1.000/1.000	1.000*/1.000	1.000/1.000
		1	1.000/1.000	1.000/1.000	1.000/1.000	1.000/1.000	1.000/1.000	1.000/1.000
		4	0.993/0.993	0.995/0.990	0.994/0.963	0.991/0.993	0.994/0.991	0.993/0.975
		9	0.859/0.862	0.843/0.834	0.862/0.723	0.851/0.859	0.858/0.847	0.851/0.776
500	0.2	0.25	0.969/0.968	0.964/0.963	0.968/0.945	0.972/0.965	0.963/0.962	0.959/0.946
		1	0.701/0.722	0.681/0.697	0.632/0.619	0.705/0.712	0.721/0.698	0.662/0.636
		4	0.306/0.299	0.310/0.282	0.283/0.227	0.289/0.295	0.305/0.288	0.279/0.244
		9	0.151/0.165	0.162/0.155	0.178/0.128	0.171/0.164	0.169/0.159	0.163/0.140
	0.5	0.25	1.000/1.000	1.000/1.000	1.000/1.000	1.000/1.000	1.000/1.000	1.000/1.000
		1	1.000/1.000	1.000/1.000	1.000/1.000	1.000/1.000	1.000/1.000	1.000/1.000
		4	0.962/0.948	0.949/0.932	0.949/0.857	0.953/0.945	0.957/0.938	0.952/0.885
		9	0.715/0.692	0.679/0.657	0.673/0.540	0.704/0.688	0.685/0.672	0.686/0.594
	0.8	0.25	1.000/1.000	1.000/1.000	1.000/1.000	1.000/1.000	1.000/1.000	1.000/1.000
		1	1.000/1.000	1.000/1.000	1.000/1.000	1.000/1.000	1.000/1.000	1.000/1.000
		4	1.000/1.000	1.000/1.000	1.000/0.998	1.000/1.000	1.000/1.000	1.000/0.999
		9	0.975/0.976	0.975/0.966	0.975/0.909	0.968/0.975	0.979/0.971	0.979/0.939

Results for a questionnaire composed of 10 items.

* The 95% confidence interval of the type I error does not contain the expected value of 5%.

We observe an impact of the gap between means of the latent variable and item difficulties (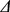) which is stronger when the gap is high (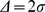). In these cases, the power obtained using CR is lower than the power of simulations. The loss of power is the highest when the variance (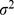 = 4 or 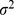 = 9) and the group effect (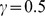 or 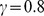) are high, the number of items 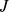 is low and the distribution of the items is normal as compared to a mixture of normal distribution of items. The loss can exceed −20% in the worst cases. For example, when 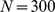, 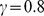, 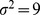, 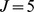, 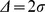 and the distribution of items was normal, the estimated power is 83.4% for the simulations and 60.3% for CR.

## Discussion

The validity of the method to estimate the standard error of group effect and to determine the power of the test of group effect in IRT using Cramer-Rao bound was investigated for a large number of situations that may be often encountered in practice. The estimated variance of group effect and power obtained using Cramer-Rao were close to the estimations from the simulations when the distributions of the latent variable and the items were overlaid (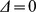). As expected, the variance of group effect increased with the variance of the latent variable. This led to a decrease of the power of the test of group effect that does not differ for both methods (Cramer-Rao and simulations). The Cramer-Rao method seems to be still valid for high values of the variance of the latent variable.

However, when the gap between means of the latent variable and item difficulties (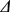) is high, we observed an inflated estimation of the variance of group effect and consequently a loss of power for CR compared to the simulations. The Cramer-Rao method seems to reach its limits for 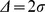 and high values of 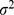 and 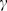. The impact of an underestimation of the power can have large consequences on the planned sample size. To achieve a power of 80% for a gap equal to 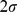 when 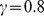 and 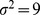, the Cramer-Rao method suggests to use 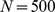 patients per group whereas 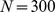 patients per group is a sufficient sample size to obtain a power of 83.4% according to the simulations. Hence, in this example, 200 patients in each group would have been unnecessarily included in the study to achieve a power of 80% using the Cramer-Rao method with a gap equals to 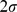. So, the choice of a questionnaire appropriate to the population at the design stage is an important issue. For example, the use of a disease-specific questionnaire in general population is not recommended as the population of the study will probably not encounter some of the symptoms strongly related to this disease. Thereby, some items evaluating the symptoms will have only few or no positive responses leading to a floor effect and an incorrect determination of the power with the Cramer-Rao method.

We recommend taking time on the choice of the questionnaire before the study. To evaluate the suitability of a questionnaire, it seems important to first check that the items composing the questionnaire intended to be used are relevant for the population of study. An item is not considered as relevant if the population will answer mainly to one of its modality only and will lead to ceiling or floor effect. When choosing a questionnaire for a study, one has to take into account the characteristics of the population used for its former validation (type of the disease, seriousness of the pathology, …) in order to be suitable enough for the population to be studied.

At the planning stage, the parameters of the distribution of the latent variable and the item parameters have to be fixed. To do so, it is easier to rely on a pilot study or on previous articles for example. Hence, it may be possible to evaluate if a gap between the mean of the latent variable and the mean of the items distribution is likely to occur.

Despite all the precautions taken at the planning stage, a gap can be observed at the analysis stage. Unfortunately, the Cramer-Rao method would have underestimated the power in this case. Consequently, the number of subjects to be included in the study would have been overestimated which raise ethics and financial problems. Given the results, it does not seem reasonable to use the Cramer-Rao method for a gap equals or higher than 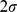. In fact, a gap equals to 2 standard deviations seems to already reflect a poorly suitable questionnaire, a generic questionnaire assessing health-related quality of life of a seriously ill population for example. However, the Cramer-Rao performs well in a large number of situations and can handle a moderate gap between the distributions of the latent variable and the items (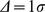).

We observed a slight impact between the quite regularly spaced items (normal distribution) and the irregularly spaced items (mixture of normal distributions) on the variance and the power when the gap was high (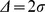). The normal distribution gave higher estimations of variance and so lower power than the mixture of normal distributions. This effect increased with the gap. It could be explained by the fact that, in the way the data were simulated in our study, the items coming from the mixture of distributions covers a wider part of the latent variable distribution as shown in Figure 1. Furthermore, when the latent variable and items distributions are not overlaid (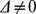), the easiest item coming from the normal distribution (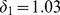 in Figure 1 (subfigure C) for example) is more on the right of the latent variable distribution than the easiest item coming from the mixture of distributions (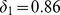 in Figure 1 (subfigure D)). Therefore, the floor effect, resulting from the gap, occurs at a lowest level of 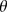 for the normal distribution than for the mixture of distributions. And so, the floor effect has more impact on the variance and power obtained using item parameters coming from the normal distribution. As this effect is linked with the simulation process, it can’t be interpreted as an impact of the regularity of the items on the performance of the Raschpower method.

Beyond the impact of items and variance of the latent trait, the effect of the sample size, the number of items and the group effect were also studied. Their values were chosen to reflect what is frequently encountered in practice in health studies. However, some assumptions had to be made to perform the simulation study. Instead of the Rasch model, another IRT model for dichotomous items could be considered such as the 2-PLM [20] or the OPLM [21]. These models are more complex than the Rasch model in the sense that they include item discriminations in addition to item difficulties. The variance using Cramer-Rao could probably be estimated with the same efficiency by adapting the formula and fixing the item discrimination to known values as made for the item difficulties.

The estimation of the variance and the determination of the power are based on the expected planned values that are fixed. This is usual at the design stage but it can turn out to be problematic if no previous studies can provide some information on the values of the parameters. If the planning values are far from the estimated values in the study at the analysis stage, the variance could be incorrectly estimated and the power for a determined sample size could then not be achieved. It seems important to further study the impact of misspecifications in the choice of the planning values on the performance of the Cramer-Rao method. The robustness of this method when some of the assumptions on the model are violated should also be evaluated to identify settings where the method should or should not be used.

For now, the main limitation of the Cramer-Rao method is that the variance can only be estimated in the frame of Patient-Reported Outcomes evaluated with dichotomous items in a cross-sectional setting. Two major developments seem to be necessary to make this method applicable in almost all studies in health sciences. First, the method should be able to deal with polytomous items. The estimation of the variance can be based on the partial-credit model [22] or the rating-scale model [23], which are extensions of the Rasch model for this type of items. The introduction of such models will lead to a more complex procedure of estimation as the number of parameters will increase with the number of modalities of the items. Second, the study of the evolution of a criteria is often of interest in health sciences. Patients’ evolution of PRO through time are often evaluated in longitudinal studies. The validity of the Cramer-Rao method in this context has to be studied as the correlated measures of patients bring into play a more complex model than in cross-sectional studies.

The estimated variance of group effect and power obtained using Cramer-Rao were close to the estimations from the simulations in most cases. These results show that the variance using Cramer-Rao bound correctly estimates the variance of the group effect. Hence, the Cramer-Rao method can be used to determine the power of the test of group effect at design stage for two-group comparison studies including patient-reported outcomes for many situations in health sciences. The important recommendation is to choose the most appropriate questionnaire for the population. Otherwise, sample size might be misspecified by this methodological approach.
